# Reply: The classification of p53 immunohistochemical staining results and patient outcome in ovarian cancer

**DOI:** 10.1038/sj.bjc.6603742

**Published:** 2007-04-17

**Authors:** P de Graeff, J Hall, A P G Crijns, G H de Bock, J Paul, K A ten Hoor, S de Jong, H Hollema, J M S Bartlett, R Brown, A G J van der Zee

**Affiliations:** 1 Department of Gynaecologic Oncology, University Medical Centre Groningen, University of Groningen, Groningen, The Netherlands; 2 Cancer Research UK Beatson Laboratories, Centre for Oncology and Applied Pharmacology, University of Glasgow, Scotland, UK; 3 Department of Epidemiology and Statistics, University Medical Centre Groningen, University of Groningen, Groningen, The Netherlands; 4 Department of Medical Oncology, University Medical Centre Groningen, University of Groningen, Groningen, The Netherlands; 5 Department of Pathology, University Medical Centre Groningen, University of Groningen, Groningen, The Netherlands; 6 Endocrine Cancer Group, University Department of Surgery, Royal Infirmary, Glasgow, Scotland, UK

**Sir**,

We appreciate the interest of Lassus and Butzow in our report on the prognostic value of p53 immunostaining in epithelial ovarian cancer. In this study, we performed statistical analysis using two cutoff methods for aberrant p53 expression as determined by immunohistochemistry: the conventional classification, in which tumour samples showing >50% moderate or strong staining are considered to show aberrant expression and the new classification as proposed by Lassus *et al* (2003) in which tumours showing completely negative, >50% moderate and strongly positive staining are considered to show aberrant p53 staining (Lassus *et al*, 2003).

We agree that immunohistochemical staining can be interpreted in different ways that may have varying success in accurately classifying the samples in biologically meaningful groups. One of the main issues raised in the letter is that further research on the prognostic value of completely negative p53 immunostaining should be undertaken. This was indeed one of the main reasons we also included analysis of the scoring system as proposed by Lassus *et al* (2003) in this large independent sample set. For reasons of clarity and conciseness, we did not extensively present results of data analysis for both classifications in the paper (de Graeff *et al*, 2006). We chose to present mainly on the conventional p53 classification because this was our original hypothesis and we centred our results on the original questions proposed, as we did not wish to bias the reporting on the basis of the study results. However, we will now take the opportunity to present these data.

Although we agree that it may be challenging to find molecular prognostic markers with prognostic capabilities stronger than some clinical factors, we argue that it is vital to put molecular markers into a clinical context using multivariate analysis to determine their potential utility. Multivariate analysis using the ‘Lassus’ classification showed that aberrant p53 immunostaining was a significant predictor of poor overall survival (*n*=222; HR 1.7, *P*=0.035). However, making some allowance for multiple testing would means that the *P*-value of 0.035 for p53 immunostaining is of borderline statistical significance. More crucially, the results of univariate analysis described below yielded markedly inconsistent results across the Dutch and Scottish cohort, raising real questions around the reliability of negative p53 immunostaining as a prognostic factor.

Statistical analysis for the Dutch and Scottish cohort separately using the ‘Lassus’ classification showed that the proportion of tumours staining completely negative was comparable across the two patient groups (26.8% in the Scottish group and 27.1% in the Dutch group). Kaplan–Meier survival analysis for progression free and overall survival showed that while strongly positive p53 immunostaining was associated with poor survival in both cohorts, the value of completely negative staining was less clear. In univariate Cox regression analysis for the Dutch cohort (*n*=188), completely negative immunostaining was strongly associated with poor PFS and OS (HR 2.5; 95%CI 1.4–4.4; *P*=0.002 and HR 3.1; 95%CI 1.7–5.4; *P*<0.001, respectively). In contrast, in the Scottish group (*n*=328) completely negative staining did not predict PFS or OS (HR 0.87; 95%CI 0.6–1.2; *P*=0.50 and HR 0.89; 95%CI 0.6–1.3; *P*=0.89, respectively). Restricting the analysis to serous carcinomas only did not influence the outcome of statistical analysis.

One possible explanation for contrasting results of statistical analysis, regarding completely negative p53 immunostaining, is that in addition to alterations at the gene, transcriptional or translational level, completely negative immunostaining could also result from time-dependent lack of antigen preservation in formalin-fixed, paraffin-embedded tissues (Prioleau and Schnitt, 1995). Even with minimal methodological variability between the two cohorts, we could not correct for differences in tissue storage and handling. For their study, Lassus *et al* (2003) included tumour tissues collected from 1964 to 2002. It is very likely that at least part of the older tumour samples used in their study stained completely negative as a result of p53 antigen loss. Mutational analysis or assessment of antigen damage by immunohistohemical control stainings (Battifora, 1991) could aid in selecting tumours that show completely negative staining resulting from mutations or alterations at the transcriptional or translational level.

In summary, although results of statistical analysis showed that aberrant p53 staining using the classification as proposed by Lassus *et al* (2003) is a potential significant predictor of overall survival in multivariate analysis, these findings should be interpreted with caution. Definitive, reliable evidence for the possible prognostic value of (completely negative) p53 staining in ovarian cancer should be obtained from clinical trials with clearly defined inclusion criteria and methodological standardisation.
